# Optimal Timing to Surgery After Neoadjuvant Chemotherapy for Locally Advanced Gastric Cancer

**DOI:** 10.3389/fonc.2020.613988

**Published:** 2020-12-17

**Authors:** Yinkui Wang, Zining Liu, Fei Shan, Xiangji Ying, Yan Zhang, Shuangxi Li, Yongning Jia, Ziyu Li, Jiafu Ji

**Affiliations:** Key Laboratory of Carcinogenesis and Translational Research (Ministry of Education/Beijing), Gastrointestinal Cancer Center, Peking University Cancer Hospital & Institute, Beijing, China

**Keywords:** gastric cancer, neoadjuvant chemotherapy, time to surgery, survival, restricted cubic spline

## Abstract

**Background:**

The relationship between time to surgery (TTS) and survival benefit is not sufficiently demonstrated by previous studies in locally advanced gastric cancer (LAGC). This study aims to assess the impact of TTS after neoadjuvant chemotherapy (NACT) on long-term and short-term outcomes in LAGC patients.

**Methods:**

Data were collected from patients with LAGC who underwent NACT between January 2007 and January 2018 at our institution. Outcomes assessed were long-term survival, pathologic complete response (pCR) rate, and postoperative complications.

**Results:**

This cohort of 426 patients was divided into five groups by weeks of TTS. Under cox regression, compared to other groups, the 22–28 days and 29–35 days groups revealed a better OS (≤21 vs. 22–28 days: HR 1.54, 95% CI = 0.81–2.93, P = 0.185; 36–42 vs. 22–28 days: HR 2.20, 95% CI = 1.28−3.79, P = 0.004; 43–84 vs. 22–28 days: HR 1.83, 95% CI = 1.09–3.06, P = 0.022) and PFS (≤21 vs. 22–28 days: HR 1.54, 95% CI = 0.81–2.93, P = 0.256; 36–42 vs. 22–28 days: HR 2.20, 95% CI = 1.28−3.79, P = 0.111; 43–84 vs. 22–28 days: HR 1.83, 95% CI = 1.09–3.06, P = 0.047). Further analysis revealed a better prognosis in patients with TTS within 22–35 days (OS: HR 1.78 95% CI = 1.25−2.54, P = 0.001; PFS: HR 1.49, 95% CI = 1.07−2.08, P = 0.017). Postoperative stay was significantly higher in the ≤21 days group, while other parameters revealed no statistical significance (P > 0.05). Restricted cubic spline depicted the nonlinear relationship between TTS and OS/PFS.

**Conclusion:**

Patients who received surgery within 3−5 weeks experienced the maximal survival benefit without an increase in postoperative complications or lowering the rate of pCR. Further investigations are warranted.

## Introduction

Although surgery is the mainstay of curative treatment for gastric cancer (GC), neoadjuvant chemotherapy (NACT) has been increasingly employed to improve the survival rate for locally advanced diseases over the past 20 years (1–3). Advantages of NACT have been widely discussed, including increasing the R0-resection rate, eliminating micrometastases, and improving tumor-related symptoms, which ultimately contribute to survival improvements (2, 4).

To date, little is known about the optimal surgical timing after completion of NACT in advanced gastric cancer (LAGC). In clinical practice, primary tumor resection is usually scheduled 4−6 weeks after NACT based on clinical trials protocols (1, 5). Studies of other gastrointestinal neoplasms such as esophageal and rectal cancer found a better survival benefit or pathological response rate in patients with longer time to surgery (TTS) (6–9). Comparable studies in GC patients have shown no effect on OS or DFS, while the improvement in pathologic complete response (pCR) is significantly higher in patients with longer TTS (10, 11). However, the conclusion regarding pathological improvement was based on a retrospective study and a relatively small sample size with only 17 of 176 patients in the >6 weeks group. On the other hand, recent studies on other non-gastrointestinal tumors favor a shorter TTS (12–14). There are theoretical concerns that extended TTS may allow local tumor progression or metastasis.

Another essential aspect that must be considered is the potential impact of extended TTS on patient anxiety (15–17). It is now recommended in some countries that patients can stay on NACT if responding to and tolerating treatment to deal with the delays of surgical treatment (18). However, the best time interval for surgery after NACT is still in the mists.

For the reasons outlined above, this retrospective study aimed to investigate the impact of TTS after NACT on survival benefit and short-term outcomes in patients with LAGC, and to establish whether there was a clear TTS interval that optimized survival and perioperative outcomes.

## Methods

### Study Patients

After obtaining approval from the Peking University Cancer Hospital Ethics Committee, we retrospectively selected patients who were diagnosed with GC from January 1, 2007, to January 1, 2018, at the Peking University Cancer Hospital and Institute.

The inclusion criteria included: (1) proven diagnosis of gastric adenocarcinoma by endoscopic biopsy prior to surgery; (2) complete clinicopathological data recorded; (3) no signs of distant metastasis at first visit; (4) patients had undergone NACT before surgery; and, (5) curative gastrectomy was performed at our center.

The exclusion criteria included: (1) patients had received chemotherapy regimens other than SOX (S-1 plus oxaliplatin) or CapeOX (capecitabine plus oxaliplatin), or had switched to other regimens during NACT; (2) incomplete surgery information; (3) operation with R1/R2 resection or peritoneal lavage fluid indicating positive peritoneal cytology (CY1) or peritoneal dissemination (P1); (4) patients received intraperitoneal chemotherapy or hyperthermia intraperitoneal chemotherapy; (5) patients received neoadjuvant radiotherapy or targeted therapy before surgery; (6) patients with non-adenocarcinoma postoperative histological findings; (7) patients without D2 lymphadenectomy; (8) inadequate clinical information and unevaluable TTS data; (9) TTS exceeded 84 days (**Figure S1**).

### Regimen and Radical Surgery

Preoperative chemotherapy according to the CapeOX or SOX regimen was performed with one to six 3-week cycles. Patients with CapeOX received oral capecitabine (1,000 mg/m^2^ twice daily on days 1–14 of each cycle) plus intravenous oxaliplatin (130 mg/m^2^ on day 1 of each cycle) every 3 weeks. Patients using SOX were given S-1 orally (twice daily on days 1–14 of each cycle) plus intravenous oxaliplatin (130 mg/m^2^ on day 1 of each cycle) every 3 weeks. The dosage of S-1 was calculated according to body surface area (BSA): BSA < 1.25 m^2^, 80 mg/day; 1.25 m^2^ ≤ BSA < 1.5 m^2^, 100 mg/day; BSA > 1.5 m^2^, 120 mg/day. When a patient was diagnosed with LAGC by endoscopic biopsy plus enhanced computed tomography (CT) and/or laparoscopic examination, treatment selection was decided and performed by our multidisciplinary team, including the choice of NACT, the initial treatment protocols, dosage reduction or withdrawal in cases of severe adverse events during chemotherapy, and evaluation for surgery after the completion of NACT or after the termination of NACT because of adverse events or tumor progression. Generally, the antitumor effect was evaluated per two cycles using abdominal CT. The therapy was prematurely terminated in cases of disease progression, and gastrectomy would be applied for resectable cases. Otherwise, gastrectomy or continued NACT was considered after obtaining informed consent and approval from patients. Subtotal or total gastrectomy plus D2 lymphadenectomy was performed. Methods of anastomosis included Billroth-I, Billroth-II with Braun, Roux-en-Y, or jejunal interposition reconstruction. Roux-en-Y reconstruction was widely performed in total gastrectomy, while for distal gastrectomy, Billroth II reconstruction was more preferred. All procedures were carried out by two chief surgeons (Z.Y.L and J.F.J) of the same experienced surgical team, following the Japanese Gastric Cancer Association (JGCA) guideline (19).

### Data Collection

The entry of clinical information on the eligible patients was completed by two independent researchers (Z.N.L. and Y.K.W.) between September 2019 and January 2020. Patient eligibility and data inconsistencies were determined by two investigators, with a third researcher needed when disagreement occurred. The following data were extracted from patients’ medical records: age, body mass index, gender, American Society of Anesthesiologists score, ECOG Performance Status, comorbidities, tumor location, tumor diameter (on short axis), differentiation grade, vascular involvement, posttherapy pathological (yp) T and N stage, type of resection, type of reconstruction, intraoperative blood loss, operative time, number of retrieved lymph nodes, postoperative stay, complications, total NACT cycles, date of surgery, date of NACT initiation, date of NACT completion, date of progression or recurrence, and date of last follow-up. All included patients were staged according to the eighth edition AJCC cancer staging manual (20). Survival-related data, including overall survival (OS) and progression-free survival (PFS), were also collected. OS was calculated from the initiation of NACT to the date of death or the most recent follow-up. PFS was determined from the initiation of NACT to any cause of recurrence or metastasis, the date of death if there was no clear recurrence of metastasis, or the last follow-up. The OS and PFS values were recorded in months. Postoperative complications were classified according to the Clavien-Dindo classification system (21). Complications included fever of unknown origin, abdominal infection or abscess, hemorrhage or transfusion, ascites, anastomotic leak, ileus, gastroparesis, lymphatic leak, and other systematic complications. Ascites was graded by the International Ascites Club System and was only recorded in patients over moderate ascites (22). The TTS was defined as the date from the end of the last cycle of NACT to radical surgery.

### Histopathology Analysis

All pathological examinations were undertaken by two experienced gastrointestinal pathologists, who were blinded to the group assignment. Efficacy was assessed using the pCR (ypT0N0) rate according to the National Comprehensive Cancer Network (NCCN) Guidelines (23).

### Follow-Up

Follow-ups were conducted quarterly *via* telephone, and if the patient (or contact person) could not be reached in three attempts, the patient was considered to be lost.

### Time to Surgery Designation

The TTS was calculated from the date of the end of the last cycle of NACT to the date of operation. This variable was categorized into the following groups: (a) ≤21 days, (b) 22−28 days, (c) 29−35 days, (d) 36−42 days, and (e) 43−84 days. The selection of TTS categories were based on several considerations: (1) We chose to have as larger groups as possible so as to detect a trend in the survival probability by TTS which might also provide information for further analysis (2) allocation of the medial groups were chosen at 7-day intervals from 21 days to 42 days by which should become more appealing to the real clinical practice (3) to maintain an adequate size and to avoid the loss of statistical power, ≤21 days and 43–84 days were set as the first and the latest boundaries (4) additionally, the latest 43–84 days group could be compared with previous studies that generally evaluate TTS over 6 weeks.

### Statistical Analysis

Continuous variables were summarized as the mean ± standard or median (IQR) and were compared across groups using the Kruskal-Wallis test. Categorical variables were analyzed using the chi-squared or Fisher’s exact test. Confounding and significantly different factors were evaluated using univariate and multivariate logistic regression. Clinical and pathological factors were assessed for long-term PFS and OS using a univariate log-rank test and a multivariate Cox proportional hazard model. The covariates, type of resection and reconstruction, were excluded in multivariate analyses because of their potential correlations between tumor location (Pearson’s coefficients, r = 0.651 and r = 0.401, respectively). We assessed nonlinear relationship between TTS and OS (or PFS) using restricted cubic spline (RCS) Cox regression with four internal knots placed at 5th, 35th, 65th, 95th centiles, as suggested by Harrell (24). The association is assumed to be nonlinear if the spline coefficients differ significantly from each other based on the Wald test for linearity. Bootstrap methods, using 1,000 sets of bootstrap sampling weights, were used to determine 95% CIs around the inflection point. Tumor or treatment characteristics that achieved a P-value < 0.10 in univariate analysis were included in the multivariate analysis. For all analyses, P < 0.05 was considered statistically significant. Bonferroni correction was applied when comparing more than two groups. Statistical analyses were performed using SE STATA (Stata Statistical Software, release 15.1; Stata Corp., College Station, TX, USA).

## Results

### Patient Demographic Information

A total of 426 patients with LAGC were included in the study. Forty-nine (11.50%) had TTS ≤ 21 days, 93 (21.83%) had TTS 22−28 days, 108 (25.35%) had TTS 29−35 days, 84 (19.72%) had TTS 36−42 days, and 92 (21.60%) had TTS 43−84 days. All patients fulfilled at least one completed cycle of NACT before radical surgery (median, 2 cycles; range, 1−6 cycles). The median follow-up was 43.5 months (range, 3−119 months) and the median TTS was 33 days (range 7−84 days). The distribution of TTS is displayed in **Figure S2**. Baseline characteristics were similar for groups divided according to TTS (**Table 1**).

**Table 1:** Demographic and clinicopathologic characteristics.

Characteristics	Total	Time From End of Neoadjuvant Therapy to Surgical Procedure, d					*P*
		≤21d	22-28d	29-35d	36-42d	43-84d	
No. of patients	426	49	93	108	84	92	
Age, median (IQR), y	60 (54–66)	61 (57–66)	60 (53–66)	59.5 (54–64)	61.5 (52.5–66.5)	63 (56–67.5)	0.099
BMI, median (IQR), (kg/m^2^)	23.74 (21.47–25.83)	23.88 (21.08–26.04)	23.51 (21.46–25.26)	23.71 (21.84–25.95)	24.11 (21.78–26.)	23.45 (21.30–25.54)	0.703
Male	327 (76.76)	36 (73.47)	71 (76.34)	82 (75.93)	65 (77.38)	73 (79.35)	0.950
ASA score							0.331
1	24 (5.63)	3 (6.12)	3 (3.23)	10 (9.26)	2 (2.38)	6 (6.52)	
2	345 (80.99)	43 (87.76)	77 (82.80)	83 (76.85)	67 (79.76)	75 (81.52)	
3	57 (13.38)	3 (6.12)	13 (13.98)	15 (13.89)	15 (17.86)	11 (11.96)	
4	0 (0.00)	0 (0.00)	0 (0.00)	0 (0.00)	0 (0.00)	0 (0.00)	
ECOG							0.392*
0	316 (74.18)	36 (73.47)	63 (67.74)	85 (78.70)	61 (72.62)	71 (77.17)	
1	100 (23.47)	11 (22.45)	28 (30.11)	22 (20.37)	19 (22.62)	20 (21.74)	
2	9 (2.11)	2 (4.08)	2 (2.15)	0 (0.0)	4 (4.76)	1 (1.09)	
3	1 (0.23)	0 (0.0)	0 (0.0)	1 (0.93)	0 (0.0)	0 (0.0)	
Comorbidities	133 (31.22)	12 (24.49)	32 (34.41)	34 (31.48)	26 (30.95)	29 (31.5)	0.829
Location							0.185
Upper	134 (31.46)	23 (46.94)	25 (26.88)	28 (25.93)	23 (27.38)	35 (38.04)	
Middle	62 (14.55)	3 (6.12)	16 (17.20)	17 (15.74)	17 (20.24)	9 (9.78)	
Lower	211 (49.53)	21 (42.86)	47 (50.54)	60 (55.56)	39 (46.43)	44 (47.83)	
Diffuse	19 (4.46)	2 (4.08)	5 (5.38)	3 (2.78)	5 (5.95)	4 (4.35)	
Diameter (cm)							0.642
≤2	249 (58.45)	22 (44.90)	53 (56.99)	67 (62.04)	52 (61.90)	55 (59.78)	
2-5	138 (32.39)	20 (40.82)	33 (35.48)	33 (30.56)	24 (28.57)	28 (30.43)	
≥5	39 (9.15)	7 (14.29)	7 (7.53)	8 (7.41)	8 (9.52)	9 (9.78)	
Differentiation							0.280
Well	11 (2.58)	1 (2.04)	3 (3.23)	3 (2.78)	3 (3.57)	1 (1.09)	
Moderate	217 (50.94)	29 (59.18)	39 (41.94)	61 (56.48)	36 (42.86)	52 (56.52)	
Poor	198 (46.48)	19 (38.78)	51 (54.84)	44 (40.74)	45 (53.57)	39 (42.39)	
Signet ring	71 (16.67)	8 (16.33)	16 (17.20)	11 (10.19)	18 (21.43)	18 (19.57)	0.266
ypT							0.689
T0	32 (7.51)	3 (6.12)	6 (6.45)	7 (6.48)	10 (11.90)	6 (6.52)	
T1	46 (10.80)	3 (6.12)	9 (9.68)	15 (13.89)	14 (16.67)	5 (5.43)	
T2	76 (17.84)	9 (18.37)	15 (16.13)	21 (19.44)	13 (15.48)	18 (19.57)	
T3	112 (26.29)	15 (30.61)	25 (26.88)	28 (25.93)	19 (22.62)	25 (27.17)	
T4	160 (37.56)	19 (38.78)	38 (40.86)	37 (34.26)	28 (33.33)	38 (41.30)	
ypN							0.934
N0	196 (46.01)	23 (46.94)	41 (44.09)	52 (48.15)	42 (50.00)	38 (41.30)	
N1	85 (19.95)	10 (20.41)	21 (22.58)	19 (17.59)	16 (19.05)	19 (20.65)	
N2	65 (15.26)	8 (16.33)	16 (17.20)	19 (17.59)	9 (10.71)	13 (14.13)	
N3	80 (18.78)	8 (16.33)	15 (16.13)	18 (16.67)	17 (20.24)	22 (23.91)	
Resection type							0.766
Total	235 (55.16)	24 (48.98)	53 (56.99)	64 (59.26)	45 (53.57)	49 (53.26)	
Subtotal	191 (44.84)	25 (51.02)	40 (43.01)	44 (40.74)	39 (46.43)	43 (46.74)	
Reconstruction							0.387
Billroth-I	42 (9.86)	7 (14.29)	8 (8.60)	13 (12.04)	6 (7.14)	8 (8.70)	
Billroth-II	122 (28.64)	13 (26.53)	30 (32.26)	24 (22.22)	22 (26.19)	33 (35.87)	
Rou-en-Y	242 (56.81)	27 (55.10)	51 (54.84)	69 (63.89)	51 (60.71)	44 (47.83)	
Jejunal interposition	20 (4.69)	2 (4.08)	4 (4.30)	2 (1.85)	5 (5.95)	7 (7.61)	
Adjuvant chemotherapy							0.188
No	77 (18.08)	11 (22.45)	15 (16.13)	18 (16.67)	10 (11.90)	23 (25.00)	
Yes	349 (81.92)	38 (77.55)	78 (83.87)	90 (83.33)	74 (88.10)	69 (75.00)	
Cycle of NACT							0.523*
1	5 (1.17)	0 (0.0)	0 (0.0)	2 (1.85)	1 (1.19)	2 (2.17)	
2	241 (56.57)	32 (65.31)	45 (48.39)	61 (56.48)	52 (61.90)	51 (55.43)	
3	151 (35.45)	13 (26.53)	43 (46.24)	37 (34.26)	27 (32.14)	31 (33.70)	
4	25 (5.87)	3 (6.12)	5 (5.38)	7 (6.48)	4 (4.76)	6 (6.52)	
5	1 (0.23)	0 (0.0)	0 (0.0)	1 (0.93)	0 (0.0)	0 (0.0)	
6	3 (0.70)	1 (2.04)	0 (0.0)	0 (0.0)	0 (0.0)	2 (2.17)	

Values in parentheses are percentages unless indicated otherwise.

BMI, Body Mass Index; ASA, American Society of Anesthesiologists; ECOG, Eastern Cooperative Oncology Group; PFS, progression-free survival; OS, overall survival; TTS, time to surgery; NACT, neoadjuvant chemotherapy.

*Value calculated by Fisher exact test.

### Follow-Up Survival and Recurrence

**Figure 1:**
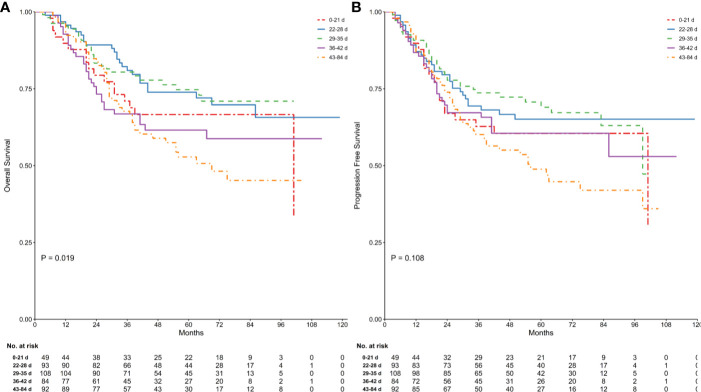
Comparison of survival in groups stratified by time between the end of neoadjuvant therapy and gastrectomy. Kaplan Meier analysis for OS (log-rank test of equality, P = 0.019; **(A)** and PFS (log-rank test of equality, P = 0.108; **(B)** were shown. Numbers below the graph indicate the number of subjects at each follow-up.

At the time of the analysis, 143 patients (33.57%) had died and 165 (38.73%) had experienced a recurrence. Kaplan–Meier curves for OS and PFS stratified by TTS are presented in **Figure 1**. The log-rank test confirmed that there were significant differences between groups in terms of OS (log-rank P = 0.019), while for PFS the result was of borderline significance (log rank P = 0.108). The Bonferroni *post hoc* test revealed OS to be significantly different between the 28−35 days and 42−84 days groups (P = 0.043), and of borderline significance between the 21−28 days and 42−84 days groups. Full details are given in **Table S1**.

The Cox proportional hazards model was formulated, adjusting for potential confounders based on P < 0.1 in ASA, ECOG, tumor location, diameter, differentiation, histological types, lymphovascular invasion, adjuvant chemotherapy, ypT and ypN stages univariate analyses (**Table 2**). Relating to TTS, results were similar to those of the univariate analyses both for OS and PFS after adjustment. The 22−28 days and 29−35 days groups revealed a better OS (HR 1.11, 95% CI = 0.64−1.93, P = 0.717) and PFS (HR 1.06, 95% CI = 0.64−1.74, P = 0.831) compared to the other groups. Patients had TTS over 42 days showed a significantly higher risk in OS (HR 1.83, 95% CI = 1.09–3.06, P = 0.022) and PFS (HR 1.62, 95% CI = 1.01−2.62, P = 0.047) compared to patients had TTS at 22–28 days. Similarly, there was statistical significance between 36–42 days and 22–28 days groups (HR 2.20, 95% CI = 1.28−3.79, P = 0.004) in OS, while in PFS the decrease of survival benefit in 36–42 days was of borderline significance (HR 1.52, 95% CI = 0.91−2.53, P = 0.111). Patients with TTS ≤ 21 days, on the other hand, revealed a poorer OS (HR 1.54, 95% CI = 0.81–2.93, P = 0.185) and PFS (HR 1.40, 95% CI = 0.78–2.52, P = 0.256) compared to patients with TTS at 22−28 days, but had no statistical significance. Additionally, tumor location, ypN stage and adjuvant chemotherapy were significantly associated with OS, while ypN was the only factor that showed statistically significant correlation with PFS between groups other than TTS.

**Table 2:** Univariate and multivariate analyses for overall survival and progression-free survival using a Cox proportional hazards model.

Variables	OS				PFS			
	Univariate	*P*	Multivariate	*P*	Univariate	*P*	Multivariate	*P*
	Hazard ratio*		Hazard ratio*		Hazard ratio*		Hazard ratio*	
Age (years)								
≤60	1.00				1.00			
>60	1.19 (0.86–1.65)	0.295			1.12 (0.82–1.52)	0.475		
BMI (kg/m^2^)								
≤23.9	1.00				1.00			
>23.9	0.86 (0.62–1.19)	0.358			0.92 (0.68–1.25)	0.584		
Gender								
Male	1.00				1.00			
Female	1.18 (0.81–1.72)	0.397			1.02 (0.71–1.47)	0.898		
ASA score								
1	1.00				1.00		1.00	
2	1.90 (0.77–4.64)	0.161			2.29 (0.94–5.59)	0.069	1.76 (0.69–4.48)	0.233
3	1.73 (0.65–4.61)	0.273			2.18 (0.83–5.72)	0.113	1.83 (0.66–5.04)	0.244
ECOG								
0	1.00				1.00		1.00	
1-3	1.31 (0.92–1.86)	0.137			1.43 (1.04–1.99)	0.028	1.37 (0.97–1.95)	0.074
Comorbidities								
No	1.00				1.00			
Yes	1.09 (0.77–1.55)	0.617			1.15 (0.83–1.59)	0.386		
Location								
Upper	1.00		1.00		1.00		1.00	
Middle	0.81 (0.45–1.46)	0.480	0.94 (0.51–1.74)	0.845	0.87 (0.52–1.46)	0.605	1.12 (0.64–1.94)	0.689
Lower	1.18 (0.81–1.73)	0.383	1.57 (1.06–2.33)	0.024	1.03 (0.72–1.46)	0.882	1.28 (0.87–1.87)	0.203
Diffuse	4.22 (2.31–7.74)	<0.001	2.56 (1.23–5.31)	0.012	4.02 (2.25–7.18)	<0.001	1.61 (0.81–3.20)	0.173
Diameter (cm)								
≤2	1.00		1.00		1.00		1.00	
2–5	1.85 (1.28–2.65)	0.001	1.11 (0.75–1.66)	0.597	1.80 (1.28–2.52)	0.001	1.14 (0.79–1.65)	0.486
>5	3.47 (2.18–5.53)	<0.001	1.16 (0.63–2.14)	0.628	3.97 (2.56–6.15)	<0.001	1.44 (0.82–2.53)	0.209
Differentiation								
Well-moderate	1.00		1.00		1.00		1.00	
Poor	1.60 (1.15–2.23)	0.005	1.02 (0.70–1.48)	0.926	1.70 (1.25–2.31)	<0.001	1.12 (0.79–1.59)	0.538
Pathology								
Non-signet	1.00		1.00		1.00		1.00	
Signet ring	2.04 (1.40–2.99)	<0.001	1.35 (0.88–2.08)	0.168	1.82 (1.26–2.62)	0.001	1.21 (0.79–1.83)	0.380
Lymphovascular invasion								
No	1.00		1.00		1.00		1.00	
Yes	3.19 (2.29–4.44)	<0.001	1.14 (0.75–1.74)	0.534	3.28 (2.41–4.46)	<0.001	1.29 (0.88–1.88)	0.192
ypT								
T0	1.00				1.00		1.00	
T1	1.27 (0.30–5.34)	0.740	1.11 (0.26–4.68)	0.888	1.11 (0.31–3.93)	0.872	1.07 (0.30–3.83)	0.916
T2	2.93 (0.87–9.92)	0.083	1.86 (0.53–6.47)	0.329	2.45 (0.84–7.15)	0.100	1.56 (0.52–4.67)	0.425
T3	4.41 (1.36–14.33)	0.014	2.39 (0.70–8.19)	0.166	3.74 (1.34–10.46)	0.012	1.97 (0.67–5.79)	0.217
T4	6.35 (2.00–20.10)	0.002	2.54 (0.75–8.59)	0.135	5.94 (2.18–16.17)	<0.001	2.33 (0.80–6.75)	0.120
ypN								
N0	1.00				1.00		1.00	
N1	1.51 (0.87-2.62)	0.140	1.15 (0.64-2.07)	0.636	1.63 (0.99-2.69)	0.053	1.24 (0.73–2.11)	0.427
N2	3.79 (2.31–6.21)	<0.001	3.17 (1.81–5.56)	<0.001	3.77 (2.38–5.98)	<0.001	2.64 (1.56–4.45)	<0.001
N3	7.41 (4.80-11.45)	<0.001	4.45 (2.51–7.89)	<0.001	8.00 (5.33–12.01)	<0.001	4.19 (2.47–7.12)	<0.001
Adjuvant chemotherapy								
Yes	1.00		1.00		1.00		1.00	
No	1.39 (0.94–2.07)	0.095	1.59 (1.06–2.40)	0.025	1.15 (0.78–1.68)	0.481		
Cycle of NACT								
≤2	1.00				1.00			
>2	0.97 (0.68–1.37)	0.861			1.01 (0.73–1.39)	0.954		
Time to surgery (days)								
≤21	1.36 (0.73–2.51)	0.333	1.54 (0.81-2.93)	0.185	1.25 (0.71–2.20)	0.430	1.40 (0.78–2.52)	0.256
22-28	1.00		1.00		1.00			
29-35	0.98 (0.57–1.69)	0.944	1.11 (0.64–1.93)	0.717	0.94 (0.58–1.53)	0.800	1.06 (0.64–1.74)	0.831
36-42	1.66 (0.98–2.82)	0.059	2.20 (1.28–3.79)	0.004	1.27 (0.77–2.08)	0.345	1.52 (0.91–2.53)	0.111
43-84	1.91 (1.17–3.13)	0.010	1.83 (1.09–3.06)	0.022	1.61 (1.03–2.54)	0.038	1.62 (1.01–2.62)	0.047

Values in parentheses are *95% confidence intervals.

BMI, Body Mass Index; ASA, American Society of Anesthesiologists; ECOG, Eastern Cooperative Oncology Group; PFS, progression-free survival; OS, overall survival; NACT, neoadjuvant chemotherapy.

**Figure 2:**
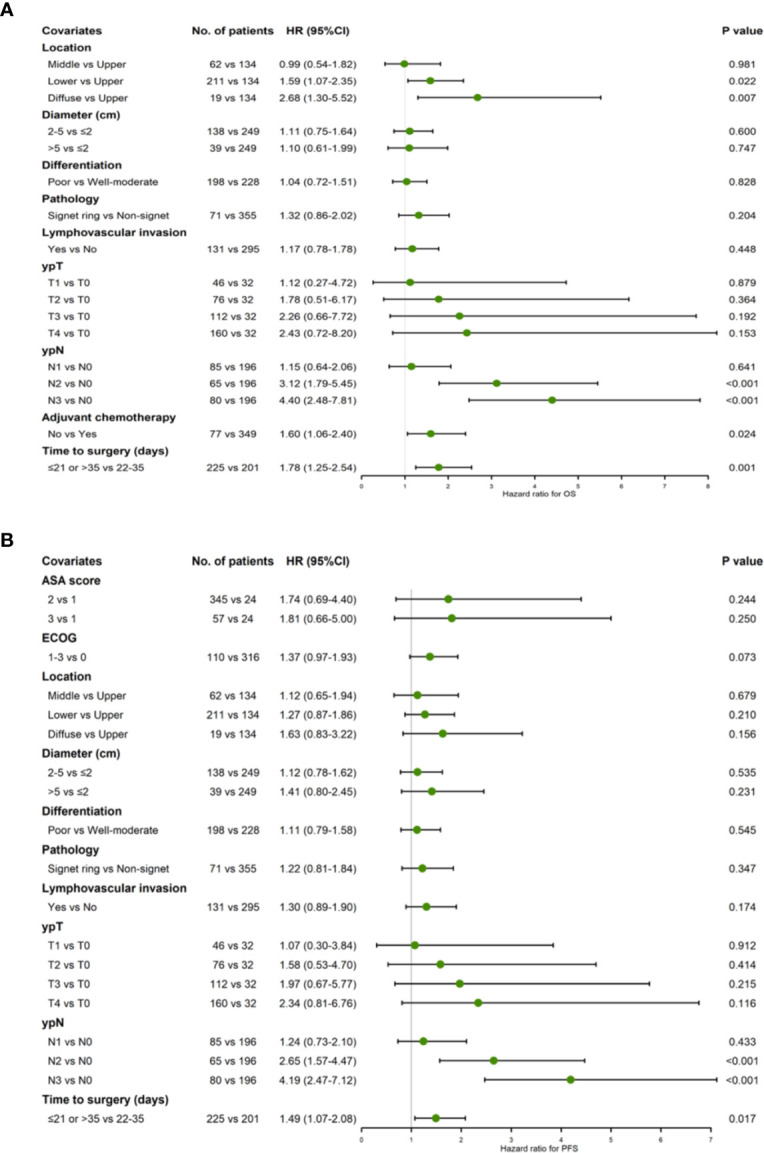
Forest plot showing hazard ratios for OS **(A)** and PFS **(B)** using a multivariable Cox proportional hazards model with binary TTS groups (22–35 days vs. ≤21/>35 days).

Since 22–28 and 29–35 groups revealed similar levels of survival benefit, the TTS were made dichotomous: TTS outside the 22–35 days intervals were taken as risk predictor of survival. The multivariate cox regression showed that TTS stepping out of the 22–35 days boundaries was significantly correlated with poorer OS (HR 1.78, 95% CI = 1.25−2.54, P = 0.002) and PFS (HR 1.49, 95% CI = 1.07−2.08, P = 0.017) prognoses (**Figures 2A, B**).

**Figure 3:**
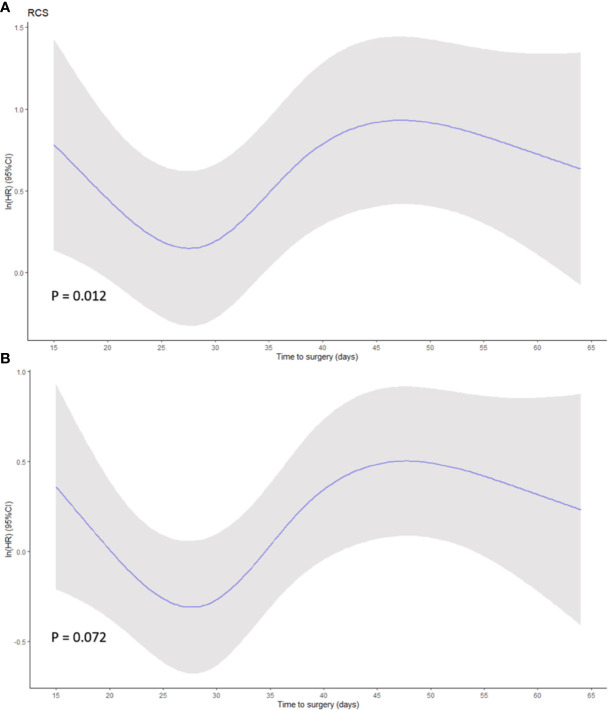
Smoothed restricted cubic splines plots of the natural logarithm of hazard ratio (HR) of **(A)** overall survival (OS) and **(B)** progression-free survival (PFS) based on Cox regression. A threshold of 28 days of time to surgery was demonstrated. Adjusted variables were location, diameter, differentiation, histological type, lymphovascular invasion, ypT stage, ypN stage, adjuvant therapy for OS model, and location, diameter, differentiation, histological type, lymphovascular invasion, ypT stage, ypN stage, ASA, and ECOG for PFS model. Y-axis demonstrates the unadjusted log hazard of mortality, and X-axis demonstrates the time to surgery in days. The greyed ribbon area reflects bounds of the 95% CI. P values were for non-linear Wald test.

Restricted cubic spline based on Cox regression were used to flexibly model the association between TTS and survival probabilities shown in **Figures 3A, B** (P = 0.012 in OS and P = 0.072 in PFS, for non-linearity). The lowest inflection point was found around 28 days of TTS, while the 24–29 days was considered the 95% reference indicating the maximum benefit of OS based on bootstrap sampling (**Figure 3A**). The confidence intervals dramatically widened when TTS exceeded 50 days, reflecting the small number of patients receiving surgery at over 50 days after NACT completion.

### Effect on pCR and Perioperative Outcomes

The rates of intraoperative and postoperative parameters are shown in **Table 3**. There was no significant difference between groups for operative time (P = 0.146), blood loss (P = 0.143), or the number of lymph nodes retrieved (P = 0.978). Regarding postoperative complications, all conditions except for lymphatic leakage showed no significant difference between groups (**Table S2A**). The rate of lymphatic leakage was higher in the ≤21 days group and showed statistical significance based on Fisher’s exact test (P = 0.012), while following a Bonferroni adjustment, no significant pairwise comparisons were observed (the lowest pairwise P-value was for ≤21 days vs. 28−35 days: 8.16% vs. 0.93%, P = 0.17). Significant correlation was observed in postoperative stay (P < 0.001): in pairwise comparison, patients in the ≤21 days group had significantly higher postoperative stay than others (vs. 21−28 days, 13 vs. 10, P < 0.001; vs. 28−35 days, 13 vs. 10, P = 0.001; vs. 35−42 days, 13 vs. 10.5, P = 0.032; vs. 42−84 days, 13 vs. 11, P = 0.036) (**Table S2B**).

**Table 3:** Perioperative parameters and pathological response regarding TTS after NACT in 426 patients.

	All patients	≤ 21d (n = 49)	21–28d (n = 93)	28–35d (n = 108)	35-42d (n = 84)	42–84d (n = 92)	*P*
Hospital stay (days)*	10 (9,14)	13 (10, 16)	10 (9, 12)	10 (9, 13)	10.5 (9,14)	11 (9,14)	<0.001
Operative time (min)*	203 (172, 239)	205 (171, 240)	215 (179, 248)	198.5 (166, 227.5)	207.5 (186.5, 252.5)	201 (170, 229.5)	0.146
Blood loss (ml)*	100 (100, 150)	100 (100, 200)	100 (99, 150)	100 (75, 173.5)	100 (100, 200)	100 (100, 150)	0.143
The number of resected lymph nodes*	30 (23, 39)	31 (24, 36)	30 (23, 40)	30 (24.5, 39)	29.5 (22, 38.5)	30 (23, 39)	0.978
Clavien-Dindo							0.369
0	297 (69.72)	30 (61.22)	70 (75.21)	81 (75.00)	56 (66.67)	60 (65.22)	
I-II	87 (21.22)	9 (18.37)	15 (16.13)	11 (10.19)	16 (19.05)	17 (18.48)	
III-IV	60 (14.63)	10 (20.41)	8 (8.60)	16 (14.81)	12 (14.29)	15 (16.30)	
Fever for unknown origin	57 (13.90)				32 (12.26)	25 (16.78)	0.203
Abdominal infection/abscess	37 (8.69)	6 (12.24)	9 (9.68)	7 (6.48)	6 (7.14)	9 (9.78)	0.745
Hemorrhage or transfusion	21 (4.93)	2 (4.08)	2 (2.15)	6 (5.56)	6 (7.14)	5 (5.43)	0.622
Moderate to massive ascites	42 (9.86)	6 (12.24)	10 (10.75)	10 (9.26)	8 (9.52)	8 (8.70)	0.964
Anastomotic leak	26 (6.10)	6 (8.16)	6 (6.45)	4 (3.70)	5 (5.95)	7 (7.61)	0.766
Ileus	11 (2.58)	0 (0.00)	1 (1.08)	3 (2.78)	3 (3.75)	4 (4.35)	0.500
Gastroparesis	10 (2.35)	2 (4.08)	1 (1.08)	3 (2.78)	1 (1.19)	3 (3.26)	0.697
Lymphatic leakage	7 (1.64)	4 (8.16)	1 (1.08)	1 (0.93)	0 (0.0)	1 (1.09)	0.012
Unexpected reoperation	6 (1.41)	2 (4.08)	1 (1.08)	2 (1.85)	1 (1.19)	0 (0.00)	0.375
Systemic complications							
Pulmonary complications^a^	52 (12.21)	9 (18.37)	6 (6.45)	11 (10.19)	13 (15.48)	13 (14.13)	0.183
Cardiovascular complications^b^	10 (2.35)	1 (2.04)	3 (3.23)	0 (0.00)	2 (2.38)	4 (4.35)	0.228
Other vascular complications^c^	4 (0.94)	1 (0.00)	1 (1.08)	2 (1.85)	1 (1.19)	0 (0.00)	0.873
Delirium	4 (0.94)	0 (0.00)	1 (1.08)	0 (0.00)	3 (3.57)	0 (0.00)	0.056
ypT0	32 (7.51)	3 (6.12)	6 (6.45)	7 (6.48)	10 (11.90)	6 (6.52)	0.572
ypN0	196 (46.01)	23 (46.94)	41 (44.09)	52 (48.15)	42 (50.00)	38 (41.30)	0.789
pCR	29 (6.81)	3 (6.12)	5 (5.38)	5 (4.63)	10 (11.90)	6 (6.52)	0.387

Values in parentheses are percentages unless indicated otherwise.

*Values are median (IQR).

TTS, time to surgery; pCR, pathological complete response.

^a^Pulmonary complications include moderate to massive pleural effusion and pneumonia.

^b^Cardiovascular complications include atrial fibrillation and acute myocardial infarction.

^c^Others vascular complications include deep vein thrombosis and pulmonary embolism.

The pCR rate was documented in 6.81% of the entire patient sample and no statistical difference in pCR rates between groups was found (P = 0.387). Similarly, there was no significant correlation in rates of ypT0 and ypN0 tumors between groups (P = 0.572 and P = 0.789, respectively) (**Table 4**). Outcomes of univariate logistic regression were depicted in **Table 4** and showed that there were no statistically significant prognostic factors to predict pCR (P < 0.05). There was higher odds of pCR in patients whose tumors are non-signet ring morphology compared to patients with signet ring carcinoma with an approaching significance (P = 0.081). Other predictors including age, gender, NACT cycles, NACT regimen, tumor location, histology, differentiation, and various set of time interval for preoperative treatment.

**Table 4:** Univariate logistic regression analysis examining the influence of patient factors on pathologic complete response (pCR).

Values	Odds ratio (95% CI)	P value
Time to surgery (days)		
≤21	1.00	
22–28	0.87 (0.20–3.81)	0.855
29–35	0.74 (0.17–3.25)	0.694
36–42	2.07 (0.54–7.93)	0.287
43–84	1.07 (0.26–4.48)	0.926
NACT cycles		
≤2	1.00	
2–4	1.05 (0.48–2.28)	0.898
≥4	4.79 (0.47–48.72)	0.185
Histologic subtype		
Signet ring	1.00	
Non-signet	5.99 (0.80–44.79)	0.081
Differentiation subtype		
Poor	1.00	
Well-moderate	0.93 (0.44–1.96)	0.841
Tumor location		
Upper	1.00	
Medium	0.98 (0.33–2.95)	0.973
Lower	0.73 (0.32–1.69)	0.468
Diffuse	NA	
NACT regimen		
SOX	1.00	
CapeOX	0.92 (0.41–2.09)	0.850
Time span between		
NACT initiation to completion	0.99 (0.97–1.01)	0.498
NACT initiation to surgery	1.00 (0.98–1.01)	0.646
Gender		
Male	1.00	
Female	0.85 (0.34–2.16)	0.737
Age (years)		
≤60	1.00	
>60	1.27 (0.60–2.72)	0.530

## Discussion

In this retrospective study of 426 patients, we investigated the association between TTS and prognostic relevance in patients with LAGC. Our results suggested that TTS within 22–35 days, namely 3–5 weeks, may be an ideal time interval that derived the maximum survival benefit. The result was fostered by RCS Cox model (95% reference interval 24–29 days, with lowest OS risk). The shorter interval did not alter the surgical safety or lower the pCR rate, but patients receiving surgery within 3 weeks led to increased postoperative stay. To the best of our knowledge, this is currently the largest study investigating the relationship between the NACT-surgery interval and long-term survival in LAGC.

Time interval to surgery after the completion of NACT is a common question asked by patients with GC, but it is a question without a definite answer. There have only been a few retrospective studies on this topic with limited sample size, and these have come to different conclusions (10, 11, 25). Liu et al. (10) first presented a retrospective study of 176 patients with GC and proposed that prolonged interval time of >6 weeks was associated with an increased rate of pCR but not with OS or DFS. The overall pCR rate was 22.7%, including 24.32% (27/111), 12.50% (6/48), and 41.18% (7/17) in <4 weeks, 4−6 weeks, and >6 weeks groups, respectively (10). However, the notable rate of pCR might require further investigation as it is recognized that pCR rate is relatively low in gastric or esophagogastric junction cancer after NACT, usually no more than 10% (26, 27). Using similar methodologies and cut-off values, Wu et al. (11) reviewed 229 cases in which the NACT to surgery interval time had no impact on the survival benefit. The entire pCR rate was 7.0% in their study, including 5.71% (4/70), 5.83% (6/103), and 10.71% (6/56) in trichotomous groups.

Although the 4−6 weeks interval is adopted in some clinical trials (1, 2, 5), the evidence for this interval and delayed surgical treatment is not indicated. In our study, the relationship between TTS and survival was demonstrated by multiple groups setting, and was subsequently reorganized by dichotomous groups that revealed 3−5 weeks as the best time interval for gastrectomy after NACT. RCS Cox model was utilized to reinforce the result of our predictions coming to the 24–29 days with maximum survival benefit. We also discovered the trend toward decreased HR when the TTS was over 50 days; however, this might also be due to the slightly overfitted dataset as the low number of samples at the tail of the distribution since confidence intervals also widened.

In contrast to the findings of Liu et al. (11), a relative short-term interval before surgery (3–5 weeks) was an independent protective factor for OS and PFS with no statistical pathologic improvement. Our results are contrary to previous findings relating to rectal and esophageal cancer, in which longer waiting time is believed to achieve a better pathological response (6, 8, 28–30). We propose several explanations for these discrepancies.

First, most patients with esophageal and rectal cancer receive neoadjuvant chemoradiation therapy (NACRT) rather than NACT. Despite gastric adenocarcinoma being a radioresponsive tumor, the efficacy of the preoperative treatment is not warranted (31, 32). It should be stressed that radiotherapy is targeted only at the localized cancerous area, while chemotherapy is a systemic treatment. The increased rate of pCR is only one side of the story (33, 34). The localized edema, inflammation, and fibrosis that generated by radiation may lead to intraoperative risks, technical difficulties, and postoperative morbidities that require recovery periods of months (35, 36). The acute and late toxicity of radiotherapy were another concern on the safety and efficacy of NACRT: The completion rate of the INT-0116 and CRITICS study were only 64% and 52% in patients underwent chemoradiation, respectively (37, 38). Even after completion, the late toxicity of radiation may greatly bring down patients’ quality of life and long-term survival (39, 40). The influence of NACT, on the other hand, has neither such solid evidence for a long waiting interval benefit, nor the higher toxicity rate as higher as radiation or chemoradiation (41, 42).

Second, as an LAGC is one of the most aggressive types of gastrointestinal tumors, unnecessary surgical delay could lead to tumor progression, which should be avoided (43, 44). The same conclusions were drawn in studies of ovarian and non-small cell lung cancer; these favored timely radical treatment rather than longer waiting intervals (12, 14).

Third, relating to pCR, there should be various factors taken into account. Currently, the effectiveness of chemotherapy for gastric adenocarcinoma is still limited by its moderate-to-poor chemosensitivity and frequent chemoresistance by scanty regimen settings (45). Regardless of some clinical features unknown significance, whether the patients can achieve pCR from systemic chemotherapy is more likely to depend on its histological and molecular subtypes, radiological features, and metabolic profiling (46–50). In our study, patients with signet-ring cell type revealed to be less likely to achieve pCR showing a borderline statistical significance (P = 0.081). Our result was consistent with previous findings by Heger et al. in which pCR rate for signet ring cell type were rare (51). Contrarily, the signet-ring and diffuse subtype displayed lower chemoresistance than the intestinal subtype *in vitro* evaluations (52). More evidence is needed to investigate the effectiveness of cell types or other pathological features as independent predictors for pCR or histological response (53).

Although results from the MAGIC trial and other studies have demonstrated the safety of surgery following NACT (2, 42), it is theoretically possible that fibrosis and tissue edema after NACT may increase the risk of D2 lymphadenectomy (54). In the CRITICS trial, the incidence of anastomotic leakage was 7.1%, a slightly higher rate than the 1.2%−5.0% when it compared to similar studies as it reported (55). In our study, overall complications, abdominal infection, hemorrhage, anastomotic leak, ileus, and systemic complications were comparable between the groups, indicating an acceptable anastomotic prognosis for shorter TTS. The lymphatic leakage, though the rate was higher in the ≤3 weeks group, did not show significant correlation under post-hoc correction. Similarly, surgery-relevant indexes including blood loss and operative time showed no statistical correlation, suggesting that surgery is tolerated in patients. The postoperative stay was significantly higher in the ≤3 weeks group, which might be because the higher rate of lymphatic leakage required longer patient in-hospital recovery. Currently, the difference in length of hospital stay, while statistically significant, is likely of limited clinical significance and requires further confirmation. Finally, our results may reveal that TTS does not have a strong association with the pCR rate from NACT.

There are several limitations to our study. First, this is a retrospective study conducted in a single center and patients’ surgical timing was determined according to previous experience. Reasons for surgery postponement, such as toxicity, nutritional status, economic and logistic reasons were not systematically recorded. Second, the limited cases of GC chemosensitivity made it difficult to determine the precise tumor response interval. Third, postoperative mortality was excluded in our study and perioperative mortality was not rated. However, as there were a limited number of deaths in our center in the past 20 years, possible differences between groups should be minimal (42). Finally, the conclusion that a TTS of 3−5 weeks achieved the best survival benefit should be cautiously interpreted. Limited by its retrospective nature, there was substantial clinical heterogeneity, due to which various factors may have influenced the optimal TTS and survival outcomes. These could include the NACT regimens, number of cycles of NACT, and patient nutritional assessments.

Despite these limitations, the 3–5 weeks interval should serve as a reminder for surgeons and oncologists that an optimal surgical timing should be considered after NACT in LAGC, rather than too early or too late. Further multi-center prospective randomized studies with large sample sizes are warranted to validate our findings and to provide additional information on the relationships between the TTS and survival benefit.

## Data Availability Statement

The raw data supporting the conclusions of this article will be made available by the authors upon reasonable request. Requests to access the datasets should be directed to ziyu_li@hsc.pku.edu.cn.

## Ethics Statement

The studies involving human participants were reviewed and approved by The Ethics Committee of Peking University Cancer Hospital. The patients/participants provided their written informed consent to participate in this study.

## Author Contributions

ZYL, YKW and ZNL designed the study. The acquisition of data was conducted by YKW, ZNL and YZ. The data analysis and interpretation was performed by ZNL and XJY; ZNL and YKW drafted the manuscript. All authors extensively and critically revised the manuscript before submission.

## Funding

This work was funded by Beijing Municipal Health Commission (DFL20181103 and ZYLX201701), and Clinical Medicine Plus X - Young Scholars Project, Peking University

## Conflict of Interest

The authors declare that the research was conducted in the absence of any commercial or financial relationships that could be construed as a potential conflict of interest.

## Supplementary Material

Supplementary Figure 1 Selection of patients for inclusion.

Supplementary Figure 2 Histogram of number of patients and density underwent gastrectomy on each waiting time. The median is indicated in each case by the vertical dashed line.

Supplementary Figure 3 Kaplan-Meier curves of overall survival **(A)** and progression-free survival **(B)** according to the time interval dichotomized by grouping TTS within 22–35 days versus others.
