# Supplementary figures and images for: Optimal Timing to Surgery After Neoadjuvant Chemotherapy for Locally Advanced Gastric Cancer
Source: Front Oncol. 2020 Dec 17;10:613988. doi: 10.3389/fonc.2020.613988 (PMC7773852; doi:10.3389/fonc.2020.613988)

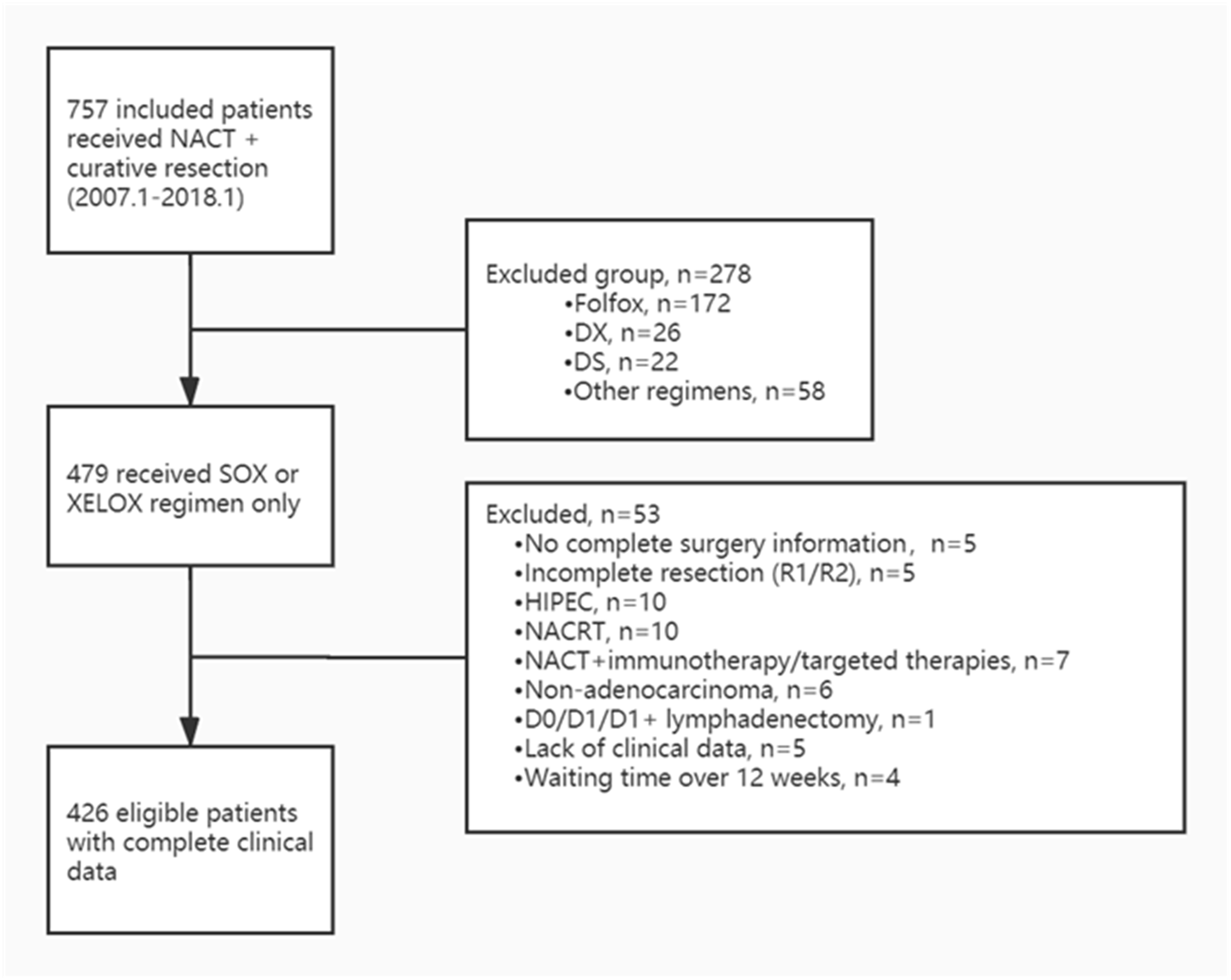

Supplement: Supplementary Figure 1 — Selection of patients for inclusion. [file Image_1.jpg]

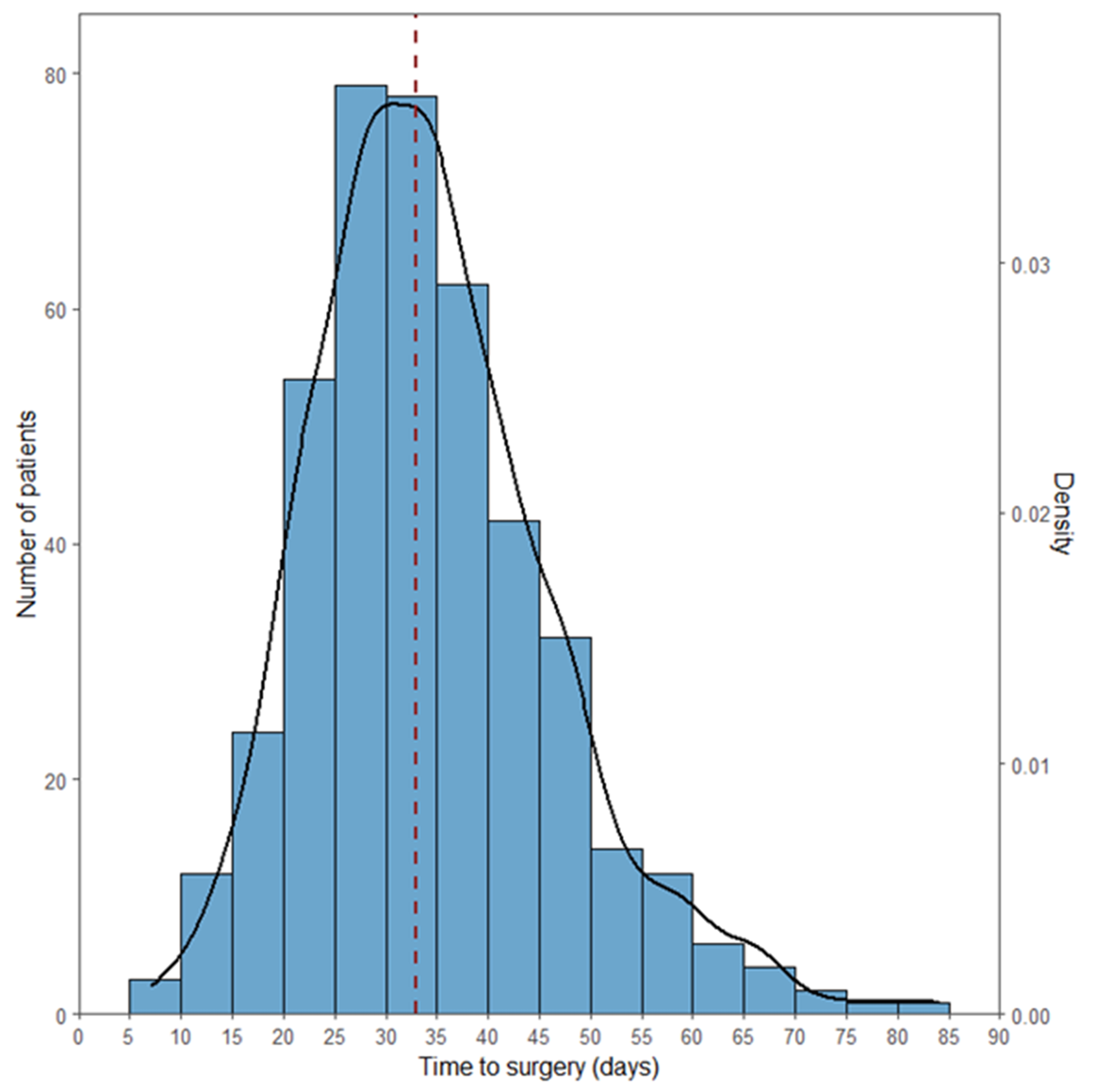

Supplement: Supplementary Figure 2 — Histogram of number of patients and density underwent gastrectomy on each waiting time. The median is indicated in each case by the vertical dashed line. [file Image_2.jpg]

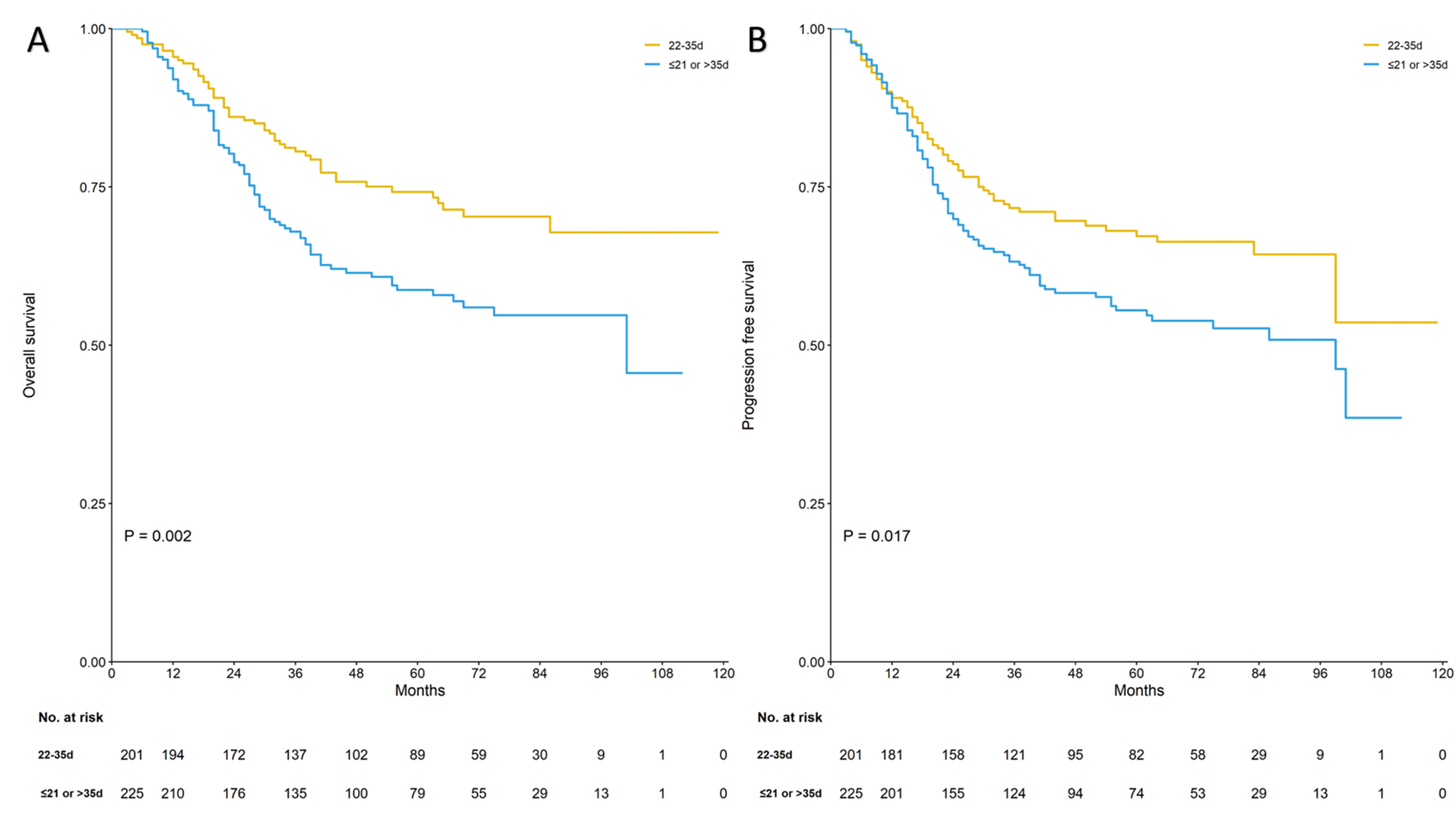

Supplement: Supplementary Figure 3 — Kaplan-Meier curves of overall survival (A) and progression-free survival (B) according to the time interval dichotomized by grouping TTS within 22–35 days versus others. [file Image_3.jpg]
