# Supplementary material for: Optimal Timing to Surgery After Neoadjuvant Chemotherapy for Locally Advanced Gastric Cancer
Source: Front Oncol. 2020 Dec 17;10:613988. doi: 10.3389/fonc.2020.613988 (PMC7773852; doi:10.3389/fonc.2020.613988)
Supplement: Supplementary file 4 [file Table_1.docx]

Table S1A P values of the pairwise log-rank test for OS with post hoc Bonferroni adjustment

|  | P values of the pairwise log-rank test | | | |
| --- | --- | --- | --- | --- |
| Groups | ≤21 days | 21-28 days | 28-35 days | 35-42 days |
| 21-28 days | 1.000 |  |  |  |
| 28-35 days | 1.000 | 1.000 |  |  |
| 35-42 days | 1.000 | 0.576 | 0.479 |  |
| 42-84 days | 1.000 | 0.060 | 0.043 | 1.000 |

Table S1B P values of the pairwise log-rank test for PFS with post hoc Bonferroni adjustment

|  | P values of the pairwise log-rank test | | | |
| --- | --- | --- | --- | --- |
| Groups | ≤21 days | 21-28 days | 28-35 days | 35-42 days |
| 21-28 days | 1.000 |  |  |  |
| 28-35 days | 1.000 | 1.000 |  |  |
| 35-42 days | 1.000 | 1.000 | 1.000 |  |
| 42-84 days | 1.000 | 0.306 | 0.109 | 1.000 |

Table S2A Inter-group comparison of lymphatic leakage with Bonferroni adjustment of p-values for post hoc test after Fisher’s exact test.

|  | P values of the pairwise Fisher’s exact test | | | |
| --- | --- | --- | --- | --- |
| Groups | ≤21 days | 21-28 days | 28-35 days | 35-42 days |
| 21-28 days | 0.480 |  |  |  |
| 28-35 days | 0.330 | 1.000 |  |  |
| 35-42 days | 0.170 | 1.000 | 1.000 |  |
| 42-84 days | 0.500 | 1.000 | 1.000 | 1.000 |

Table S2B Inter-group comparison of postoperative stay done by Dunn’s test with Bonferroni adjustment of p-values for post hoc test after Kruskal-Wallis test.

|  | P values of the Dunn’s test test | | | |
| --- | --- | --- | --- | --- |
| Groups | ≤21 days | 21-28 days | 28-35 days | 35-42 days |
| 21-28 days | <0.001 |  |  |  |
| 28-35 days | 0.001 | 1.000 |  |  |
| 35-42 days | 0.032 | 0.298 | 1.000 |  |
| 42-84 days | 0.036 | 0.214 | 1.000 | 1.000 |
